# The cingulum as a marker of individual differences in neurocognitive development

**DOI:** 10.1038/s41598-019-38894-z

**Published:** 2019-02-19

**Authors:** Joe Bathelt, Amy Johnson, Mengya Zhang, Duncan E. Astle

**Affiliations:** MRC Cognition & Brain Sciences Unit, University of Cambridge, Cambridge, United Kingdom

## Abstract

The canonical approach to exploring brain-behaviour relationships is to group individuals according to a phenotype of interest, and then explore the neural correlates of this grouping. A limitation of this approach is that multiple aetiological pathways could result in a similar phenotype, so the role of any one brain mechanism may be substantially underestimated. Building on advances in network analysis, we used a data-driven community-clustering algorithm to identify robust subgroups based on white-matter microstructure in childhood and adolescence (total N = 313, mean age: 11.24 years). The algorithm indicated the presence of two equal-size groups that show a critical difference in fractional anisotropy (FA) of the left and right cingulum. Applying the brain-based grouping in independent samples, we find that these different ‘brain types’ had profoundly different cognitive abilities with higher performance in the higher FA group. Further, a connectomics analysis indicated reduced structural connectivity in the low FA subgroup that was strongly related to reduced functional activation of the default mode network. These results provide a proof-of-concept that bottom-up brain-based groupings can be identified that relate to cognitive performance. This provides a first demonstration of a complimentary approach for investigating individual differences in brain structure and function, particularly for neurodevelopmental disorders where researchers are often faced with phenotypes that are difficult to define at the cognitive or behavioural level.

## Introduction

Differential psychology is an influential strand of modern psychology, concerned with identifying dimensions upon which individuals differ. This approach has been applied at different points across development, from childhood to old adulthood, and has been central to our understanding of typical and atypical behaviour and psychopathology^1,2^. Our understanding of the brain mechanisms associated with these differences is based almost entirely on mapping them via correlations with brain differences. This has established many consistent and significant brain-behaviour relationships in health and disease. But understanding neurobiology only through our prior understanding of cognition or behaviour has drawbacks. Firstly, our understanding of the neurobiology is entirely constrained by the choice of cognitive measures. Secondly, brain-behaviour relationships established using this logic are difficult to replicate^3,4^. This partly reflects the dependency on task selection, which differs across research groups and may only partially tap the dimensions of interest. But more critically, multiple aetiological pathways could lead to disorders with superficially similar phenotypes^5,6^. In short, grouping purely by cognitive or behavioural phenotype is no guarantee of common underlying neurobiology.

At present, there are few alternatives to complement this standard approach. Applying a data-driven clustering method that has proven useful for identifying consistent clusters of behavioural or cognitive performance^7–11^, the current study shows that it is possible to group individuals by brain organization itself rather than by cognitive or behavioural phenotype. That is, we identified stable subgroups of individuals with similar profiles of white matter microstructure across 20 major white matter tracts using a consensus community clustering algorithm. In doing so, we hoped to identify the elements of neurobiology most critical for dividing individuals into their respective subgroups and their wider functional consequences. Identifying mechanisms of brain difference *a priori* has the potential to highlight key organizational principles that we may never capture by only looking for neural correlates of messy pre-defined phenotypes.

## The Importance of White Matter in Cognitive Development

White matter makes up around half of the human brain and plays a critical role as the main conductor of neural signalling. White matter also shows prolonged post-natal changes that extend into the third decade of life^12^ with pronounced development during mid-childhood and adolescence^13^. In humans, the method of choice for measuring differences in white matter *in vivo* is diffusion-weighted imaging (DWI). It quantifies the degree to which diffusion of water molecules is restricted by the tightly-packed parallel axons that make up white matter. Differences in DWI-derived measures have been found to relate to individual differences across a range of cognitive domains. For instance, language processing is related to the maturation of the arcuate fasciculus, a white matter tract that connects the frontal and superior temporal lobe^14^; working memory performance is associated with the integrity of the superior longitudinal fasciculus, a tract connecting frontal and parietal regions^15^; executive control is linked to the integrity of frontal and parietal connections alongside additional connections to motor control regions^16^. Differences in white matter organisation have also been identified in various neurodevelopmental disorders. For instance, dyslexia is associated with a reduced organisation in white matter pathways along the left dorsal and ventral language pathways^17^; children with Attention Deficit Hyperactivity Disorder (ADHD) show reduced white matter organisation of the corpus callosum and major tracts of the right hemisphere^18^, and reduced integrity of connections between the limbic system and frontal and temporal cortex has been reported for autism spectrum disorder (ASD)^19^. In summary, DWI measures of white matter organisation are sensitive to typical variation in cognitive abilities and show differences in common neurodevelopmental disorders.

## Using Community Detection to Identify Subgroups with Similar White Matter Organisation

Many different methods have been proposed for identifying patterns of similarity between data points^20^, i.e. clustering. These methods differ in the definition of similarity between data points, e.g. correlation or Euclidean distance, and the definition of a clusters, e.g. similar distance from a cluster centre or shared variance. There is no general consensus regarding which clustering assumptions are most appropriate for a particular application. In the absence of such guidance, the current study applied a method from network science that has proven useful for distinguishing individual differences in behavioural and cognitive profiles^7–9^. Network science provides a versatile framework for any data that can be described as a collection of entities (nodes) that share quantifiable relationships (edges)^21^. Mathematical tools can be applied for the analysis of these networks. One area of intense research is the identification of highly-connected subdivision within the network called communities in analogy to communities within a social network. The community detection approach is able to detect subgroups of participants that are the most similar to each other while being as distinct as possible from other subgroups. Network science tools for community detection have been successfully used to identify social groups in phone networks and to identify brain regions with similar function^22^. This approach has also been successfully applied to distinguish differences in behaviour within heterogeneous groups, such as subgroups of neuropsychological function in typically-developing children^8^ and subgroups of executive function-related behavioural problems in children who struggle in school^9^. In summary, community-clustering provides a promising method to identify subgroups of individuals with similar characteristics. In the current analysis, community detection was applied to detect subgroups of participants with similar microstructure in 20 major tracts.

## Participants and Methods

**Figure 1:**
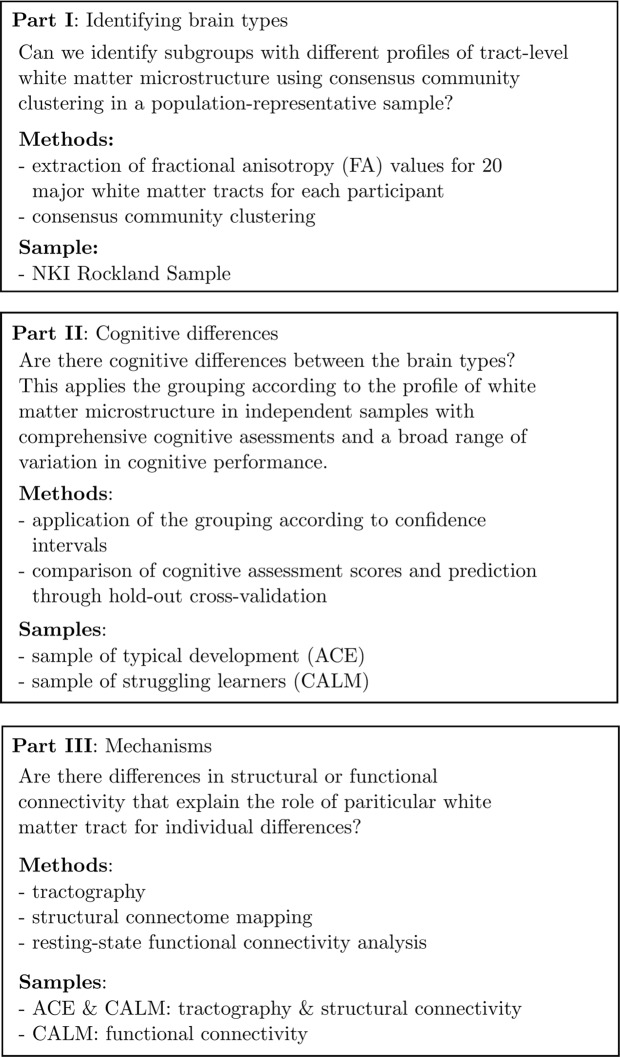
Overview of the parts of the analysis.

Please see Fig. 1 for an overview of the different parts of the analysis.

### Participants

The participants were drawn from three separate studies of development. The participants included in each study are characterized in the following sections.

#### The Nathan-Kline Institute Rockland Sample (NKI-RS)

The enhanced Nathan Kline Institute-Rockland Sample (NKI-RS) is an ongoing, institutionally-centred endeavour aimed at creating a large-scale community sample of participants across the lifespan. Details about the sample are described on the NKI website (http://fcon_1000.projects.nitrc.org/indi/enhanced/). NKI sample data is available to researchers upon request. For the current study, data from all participants who had structural imaging (T1, diffusion-weighted) and age information available was requested (date of data access: 18 July 2017). The study was approved by the NKI institutional review board and all adult and child subjects provided informed consent^23^. Original Ethical Review and consent procedures for the NKI-Rockland Sample were described as follows: “Institutional Review Board Approval was obtained for this project at the Nathan Kline Institute (Phase I #226781 and Phase II #239708) and at Montclair State University (Phase I #000983A and Phase II #000983B). Written informed consent was obtained for all study participants. Written consent and assent was also obtained from minor/child participants and their legal guardian”^23^.

#### Centre for Attention, Learning, and Memory (CALM)

For this sample, children aged between 5 and 18 years were recruited on the basis of ongoing problems in attention, learning, language and memory, as identified by professionals working in schools or specialist children’s services in the community. Following an initial referral, the CALM staff then contacted referrers to discuss the nature of the children’s problems^24^. If difficulties in one or more areas of attention, learning, language or memory were indicated by the referrer, the family were invited to the CALM clinic at the MRC Cognition and Brain Sciences Unit in Cambridge for a 3-hour assessment. This assessment included the cognitive assessments reported here. Children were excluded if they had significant problems with vision or hearing that were not corrected or if they did not have English as a native language. Written consent was obtained from parents and verbal assent from children. Families were also invited to participate in MRI scanning on a separate visit. Participation in the MRI part of the study was optional and required separate parental consent and child assent. Contra-indications for MRI were metal implants, claustrophobia, or distress during a practice session with a realistic mock MRI scanner. This study was approved by the National Health Service (NHS) Health Research Authority NRES Committee East of England (REC approval reference: 13/EE/0157, IRAS 127675) and was conducted in accordance with the Declaration of Helsinki.

#### Attention and Cognition in Education (ACE)

This sample was collected for a study investigating the neural, cognitive, and environmental markers of risk and resilience in children. Children between 7 and 12 years attending mainstream school in the UK, with normal or corrected-to-normal vision or hearing, and no history of brain injury were recruited via local schools and through advertisement in public places (childcare and community centres, libraries). Participating families were invited to the MRC Cognition and Brain Sciences Unit for a 2-hour assessment, which included the cognitive assessments reported here, and structural MRI scanning. Participants received monetary compensation for taking part in the study. This study was approved by the Psychology Research Ethics Committee at the University of Cambridge (Reference: Pre.2015.11) and was carried out in accordance with the Declaration of Helsinki. Parents or guardians provided written informed consent and children verbal assent.

### Cognitive assessments

#### Procedure

All children for whom we have cognitive data were tested on a one-to-one basis with a researcher in a dedicated child-friendly testing room at the MRC CBU. The battery included a wide range of standardized assessments of learning and cognition. Regular breaks were included throughout the session. Testing was split into two sessions for children who struggled to complete the assessments in one sitting. Measures relating a cognitive performance across different domains are included in this analysis. Tasks that were based on reaction times were not included in this analysis due to their different psychometric properties compared to the included tasks that were based on performance measures.

#### Fluid Intelligence

Fluid intelligence was assessed on the Reasoning task of the Wechsler Abbreviated Scale of Intelligence, 2^nd^ edition^25^. Both children in the CALM and ACE sample completed this assessment.

#### Working Memory

The Digit Recall, Backward Digit Recall, Dot Matrix, and Mr. X task of the Automatic Working Memory Assessment (AWMA)^26^ were administered individually. In Digit Recall, children repeat sequences of single-digit numbers presented in an audio format. In Backward Digit Recall, children repeat the sequence in backwards order. These tasks were selected to engage verbal short-term and working memory, respectively. For the Dot Matrix task, the child was shown the position of a red dot for 2 seconds in a series of four by four matrices and had to recall this position by tapping the squares on the computer screen. In the Mr X task, the child retains spatial locations whilst performing interleaved mental rotation decisions. These tasks were selected to engage visual short-term and working memory, respectively. These assessments were the same in the CALM and ACE sample.

#### Vocabulary

For the CALM sample, the Peabody Picture Vocabulary Test, Fourth Edition (PPVT-4)^27^ was used to assess receptive vocabulary knowledge. Children were required to select one of four pictures showing the meaning of a spoken word. In the ACE sample, the Vocabulary subtest of the Wechsler Abbreviated Scale of Intelligence, 2^nd^ edition^25^ was used. For this task, children had to define words that were presented verbally and visually, and correct definitions were scored.

#### Long-Term Memory

For the CALM sample, the Stories task of the Children’s Memory Scale^28^ was used to assess long-term memory. Children were read two short stories and were asked to recall these stories after a delay of 10 min. No long-term memory task was used in the ACE sample.

### MRI data acquisition

All three large-scale datasets contributed to the MRI analysis. The datasets were selected because they included similar neuroimaging data (diffusion-weighted data with a high number of directions).

#### NKI-RS

Subjects in the NKI sample underwent a scan session using a Siemens TrioTM 3.0T MRI scanner. T1-weighted images were acquired a magnetization-prepared rapid gradient echo (MPRAGE) sequence with 1 mm isotropic resolution. Diffusion scans were acquired with an isotopic set of gradients with 64 directions using a weighting factor of b = 1000 s * mm^−2^ and an isotropic resolution of 2 mm. Details about the scan sequences are described elsewhere (http://fcon_1000.projects.nitrc.org/indi/enhanced/mri_protocol.html).

#### CALM and ACE

Magnetic resonance imaging data were acquired at the MRC Cognition and Brain Sciences Unit, Cambridge U.K., on the Siemens 3 T Tim Trio system (Siemens Healthcare, Erlangen, Germany) with a 32-channel quadrature head coil. For ACE, the imaging protocol consisted of a T1-weighted anatomical sequence and a diffusion-weighted sequence. The CALM protocol also included a resting-state functional MRI (rs-fMRI) scan. The T1-weighted anatomical images were acquired using a 3D Magnetisation Prepared Rapid Acquisition Gradient Echo (MP-RAGE) sequence with 1 mm^3^ image resolution and covered the whole brain (echo time: 2.98 ms; repetition time: 2250 ms). The diffusion sequence consisted of 60 axial images that covered the whole brain with 2 mm^3^ resolution and used non-collinear directions at a weighting factor of b = 1000 s * mm^−2^ and an interleaved T2-weighted image at b = 0 s * mm^−2^ (echo time: 90 ms; repetition time: 8400 ms). For functional scans, a total of 270 T2*-weighted whole-brain echo planar images (EPIs) were acquired (time repetition[TR] = 2 s; time echo [TE] = 30 ms; flip angle = 78 degrees, 3 × 3 × 3 mm). During this scan, participants were instructed to lie still with their eyes closed and not fall asleep.

#### MRI Quality Control

Participant movement may significantly affect the quality of MRI data and may bias statistical comparisons. The quality of the diffusion-weighted and resting-state data were assessed by calculating the displacement between subsequent volumes in the sequence. Only dwi data with between-volume displacement below 3 mm and resting-state data with displacement below 0.5 mm were included in the analysis (see Table 1 for attainment). Further, all T1-weighted images and fractional anisotropy (FA) maps were visually inspected by a trained researcher (J.B.) to remove low quality scans. For ACE and CALM, several steps were taken during data acquisition to minimize participant movement. Children were instructed to lie still and were trained to do so in a realistic mock scanner prior to the actual scan.

**Table 1:** Overview of sample characteristics in the NKI, ACE, and CALM sample. The number of participants excluded because of poor quality on visual inspection (v.), an incomplete sequence (i.), or excessive movement (m. >3 mm displacement) is listed in the third column.

	n: useable structural MRI (total)	n: rejected (v./i./m.)	Gender: male-female	Age: mean (SD)	Age range
NKI	74 (84)	4/0/6	43/31	13.93 (3.164)	6.97–21.58
ACE	74 (86)	1/1/10	35/39	9.99 (1.525)	6.92–12.65
CALM	165 (206)	4/19/18	109/56	9.81 (1.191)	5.92–17.92

### Microstructural integrity of major white matter tracts

**Figure 2:**
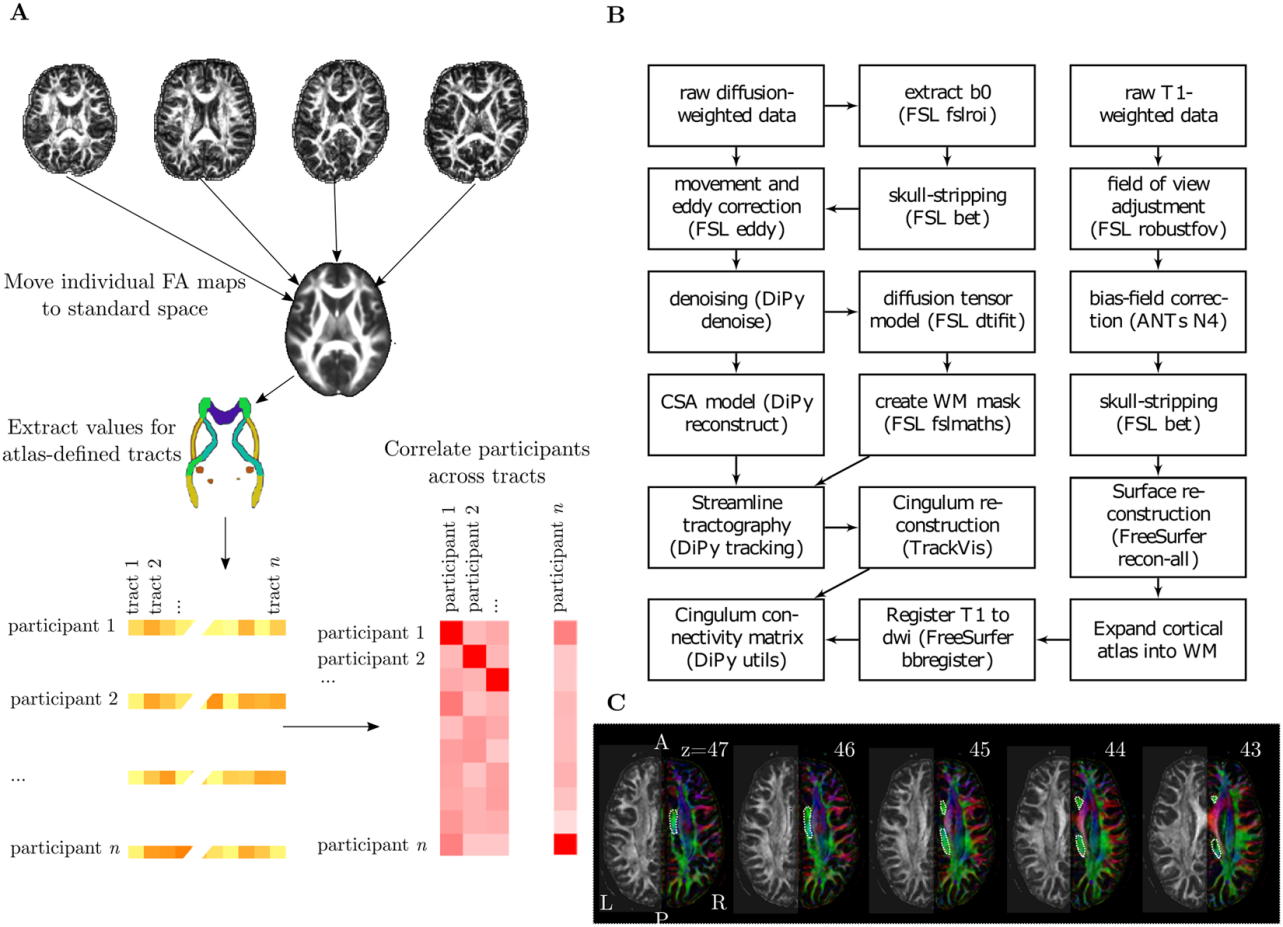
(**A**) Illustration of processing steps to obtain estimates of similarity between individuals across major white matter tracts. (**B)** Overview of processing steps to derive structural connectivity of the cingulum. (**C)** Illustration of the ROI used for tractography of the cingulum. The ROI is marked with a dotted line on consecutive FA maps coloured according to the direction of diffusion. The left side of each figure shows the FA values.

This analysis aimed to identify white matter tracts that show robust inter-individual differences during development (see Fig. 2A). Diffusion-weighted images were pre-processed to create a brain mask based on the b0-weighted image (FSL BET)^29^ and to correct for movement and eddy current-induced distortions (*eddy*)^30^. Subsequently, the diffusion tensor model was fitted and fractional anisotropy (FA) maps were calculated (*dtifit*). Images with a between-image displacement greater than 3 mm as indicated by FSL *eddy* were excluded from further analysis. All steps were carried out with FSL v5.0.9 and were implemented in a pipeline using NiPyPe v0.13.0^31^. To extract FA values for major white matter tracts, FA images were registered to the FMRIB58 FA template in MNI space using symmetric diffeomorphic image registration (*SyN*) as implemented in ANTS v1.9^32^. Visual inspection indicated good image registration for all participants. Subsequently, binary masks from a white matter atlas in MNI space^33^ were applied to extract FA values for 20 major white matter tracts^33^. An additional analysis was carried out to account for crossing fibres by calculating Generalized FA (GFA)^34^, from a constant solid angle model (details see connectome processing).

### Accounting for differences in age and gender

The current study aimed to identify important features of individual differences in brain white matter and then investigate potential cognitive differences in independent samples. In order to account for differences in the age and gender composition between samples, the current study focused on age- and gender-invariant features of white matter. To account for age- and gender-related effect, age (linear, squared) and gender were regressed from the FA values of each tract. Further, FA values were scaled to zero-mean and unit variance to account for differences in absolute FA values arising from different scanning protocols.

### Community detection

Community detection is an optimisation clustering method that aims to identify nodes within a network that are more closely connected to each other than the rest of the network. In the current analysis, the networks represented the child-by-child Pearson correlations across the FA values for the 20 white matter tracts, i.e. the correlation of FA across all tracts between children was calculated to create the child-by-child correlation matrix (see Fig. 2A).

Here, we employed the Louvain method first described by Blondel *et al*.^35^, because it has been found to provide robust community solutions in studies comparing different algorithms^36^ and because it has been successfully employed to investigate heterogeneity in cognition and behaviour before^7–9^. The current study used an extension of the Louvain method by Rubinov and Sporns^37^ that can incorporate negative edge weights as implemented in the Brain Connectivity Toolbox (https://sites.google.com/site/bctnet/ accessed August 2017). Initially, each node, here participant, is assigned to its own community. Subsequently, communities are merged across iterations if merging increases the quality index (Q), which quantifies the separation of communities and their internal consistency. The formal definition as described in Rubinov and Sporns^38^ is as follows: “The connection between nodes *i* and *j* with positive weight is ${w}_{ij}^{+}\in (0,1]$ and with negative weight is ${w}_{ij}^{-}\in [\,-\,1,0)$. The total sum of all positive connections is ${v}^{+}=\sum _{ij}\,{w}_{ij}^{+}$ and all negative connections is ${v}^{-}=\sum _{ij}\,{w}_{ij}^{-}$. The strength of the node *i* is ${s}_{i}^{\pm }=\sum _{j}\,{w}_{ij}^{\pm }$. The chance-expected within-module connection weight is ${e}_{ij}^{+}=\frac{{s}_{i}^{+}{s}_{j}^{+}}{{v}^{+}}$ for positive connections and ${e}_{ij}^{-}=\frac{{s}_{i}^{-}{s}_{j}^{-}}{{v}^{-}}$ for negative connections. ${\delta }_{{M}_{i}{M}_{j}}$ is 1 when *i* and *j* are in the same module and 0 otherwise. The quality index is defined as $Q=\frac{1}{{v}^{+}}\sum _{ij}\,({w}_{ij}^{+}-{e}_{ij}^{+})\,{\delta }_{{M}_{i}{M}_{j}}-\frac{1}{{v}^{+}+{v}^{-}}\sum _{ij}\,({w}_{ij}^{-}-{e}_{ij}^{-})\,{\delta }_{{M}_{i}{M}_{j}}$” [paraphrased quote].

Due to the non-deterministic nature of the algorithm, it may provide different solutions on different runs. Lancichinetti and Fortunato^39^ provide a consensus community method to obtain a stable solution. In an initial step, an average community solution across 100 instantiations of the community clustering algorithm is generated. Then, the community clustering is further repeated until there is not change between successive iterations.

We tested the ability of the algorithm to resolve clusters in simulated networks with a known community structure under different conditions including varying within- and between-community connection strength and with added noise. These results are available elsewhere^9^. The parameters of within- and between-community connection strength in the participant-by-participant correlation network in the current study fell within the range that can be reliably resolved according to our simulations.

To provide a comparison with an alternative clustering method, we compared the consensus community clustering solution to solutions provided by agglomerative clustering as implemented in sklearn v0.19.2^40^. Pearson correlation was used as the affinity metric for agglomerative clustering. The agglomerative clustering by itself does not optimize the number of clusters. Instead, we used the number of clusters that maximized the silhouette coefficient^41^. Further, there are several alternative linkage methods to merge clusters, specifically Ward, average, maximal, and single linkage. Across all linkage definition, the silhouette coefficient was maximal with two clusters (*k* = 2). We compared the agreement between the consensus community clustering solution with agglomerative clustering at *k* = 2 for all linkage definitions.

### Applying the community solution to other samples

In order to relate the grouping identified in the discovery sample (NKI) to other samples (ACE, CALM), we assigned participants to their age-regressed cingulum FA values fell within the 5–95%ile confidence interval of C1 or C2 in the NKI sample. Participants who fell outside of both ranges were labelled as C0. We further investigated an optimal cingulum-based split for generalizable prediction of behavioural scores. For this purpose, we employed a hold-out cross-validation procedure. The data in each sample were split into a training, a test, and a validation sample of equal size. In the test set, we assigned the participants to one of the groups identified in the discovery sample (NKI) according to the strength of their correlation with either group. The cut-off for correlation strength was tuned to maximize generalizability in the test set. Finally, we estimated how predictive these assignments were of cognitive differences in the validation set and determined the optimal percentile split for age-regressed cingulum FA. The performance of behavioural prediction was measured as the percentage of participants who were accurately predicted to perform above or below the median of the distribution based on their group assignment. For statistical analysis, we compared the performance of this behavioural prediction to a random group assignment with 1000 permutations.

### Tractography of the cingulum

To foreshadow our results, the clustering algorithm identified the cingulum as critical for determining subgroup membership. We subsequently followed this up with tractography. For the tractography, a virtual *in-vivo* dissection approach was applied^42^ (see Fig. 2B for an overview of the processing steps). For this purpose, MRI scans were converted to compressed NIfTI-1 format with the dcm2nii tool (http://www.mccauslandcenter.sc.edu/mricro/mricron/dcm2nii.html). Subsequently, the images were submitted to the DiPy v0.8.0 implementation^43^ of a non-local means de-noising algorithm^44^ to boost signal-to-noise ratio. Next, a brain mask of the b0 image was created using the brain extraction tool (BET) of the FMRIB Software Library (FSL) v5.0.8. Motion and eddy current correction was applied to the masked images using FSL routines. Finally, fractional anisotropy maps were calculated using FSL *dtifit*^45^. A constant solid angle (CSA) model as implemented in DiPy was fitted to the diffusion-weighted images with a maximum harmonic order of 8. Next, we created a whole-brain connectome through probabilistic tractography with 8 streamline seeds in all voxels with a GFA >0.1, a step size 0.5, and a maximum number of 2 crossing fibres per voxel. The cingulum in the left and right hemisphere was reconstructed in the native space of each participant by drawing a single region of interest (ROI) on 10–15 consecutive axial slices that followed the anatomy of the cingulum as described in^42^ (see Fig. 2C). To check the reliability of the cingulum tractography, two researchers performed tractography independently on 40 datasets. The spatial correlation between the density maps indicated very good inter-rater agreement (left: mean = 0.95, SE = 0.005, range = 0.86–0.990; right: mean = 0.95, SE = 0.005, range = 0.87–0.990).

### Structural connectivity of the cingulum

To estimate the structural connectivity of the cingulum, streamlines included in the cingulum tractography that intersected with cortical and subcortical ROIs were counted. The ROIs were defined using the T1-weighted anatomical images. For this purpose, the T1-weighted images were submitted to a preprocessing pipeline that adjusted the field of view (FSL *robustfov*), corrected local noise (DiPy non-local means denoising), and created a brain mask (Advanced Normalization Tools (ANTs) v1.9^46^ *BrainExtraction*). The preprocessed images were then submitted to the FreeSurfer v5.3 *recon-all* pipeline (http://surfer.nmr.mgh.harvard.edu). Regions of the Desikan-Killiany atlas^47^ with 32 cortical and 17 subcortical (amygdala, brain stem, caudate, cerebellum, hippocampus, nucleus accumbens, pallidum, putamen, thalamus) were used as ROIs. The surface-based representation of the cortical ROIs was transformed to volume representation using FreeSurfers’ *aparc2aseg* tool. To create an intersection between the ROIs and the white matter streamlines, we expended the cortical parcellation 2 mm into the subcortical white matter using an in-house implementation. Next, we calculated the transformation between the T1-weighted anatomical image and the diffusion-weighted image using FreeSurfers’ *bbregister* and applied the transformation to the ROI volume to move the ROIs into the diffusion space. For the connectome construction, we calculated the number of streamlines intersection both ROIs in each pairwise combination of ROIs. We averaged streamlines starting and ending in each ROI to create a symmetric adjacency matrix.

In order to minimize false positive connections, the streamline density matrices were thresholded so that only connections with more than 5 streamlines were retained. Further, the matrices were consensus-thresholded to only contain connections that were found in at least 60% of the sample^48^. Subsequently, the streamline density matrices were log_10_-scaled.

Individual connections that consistently differed between the groups were identified through hold-out cross-validation in a dataset combining tractography results from the ACE and CALM sample (n = 239). The dataset was randomly split into a training (80%, n = 188) and a test set (20%, n = 47). Within the training set, connections that showed a significant difference between cingulum-defined groups on a Mann-Whitney-U test that were consistently observed across 4 random split of the training data (n = 47) were selected. Subsequently, differences between groups in the strength of the selected connections were checked in the hold-out test set. Only connections that showed consistent differences in the training and test set are presented in the Results section.

### Default mode network activation

Resting-state functional MRI data were processed to obtain functional activation within the default mode network. Only participants who completed the full resting-state sequence, had full coverage of the brain in the resting-state sequence, and also had useable T1-weighted data were included in the analysis. For motion and eddy current correction, volumes were co-registered to the middle volume using *mcflirt*^49^. For quality control, the frame-wise displacement was calculated as the weighted average of the rotation and translation parameters^50^ using *fsl_motion_outliers*. Participants with a frame-wise displacement above 0.5 on this measure were excluded from the analysis. Complete data was available for 124 participants (78 male, Age: mean = 10.00, SE = 0.196, group1: n = 59, group2: n = 58, no group: n = 6). There was no significant difference in age between the sample included in the rsfMRI analysis and the sample included in the main analysis (t(284) = −0.74, p = 0.458). The samples did not differ on any of the cognitive measures (MR: t = −0.85, *p* = 0.396, Vocab: t = −0.80, *p* = 0.423; DR: t = −0.73, *p* = 0.465; DM: t = −0.62, *p* = 0.539; BR: t = −0.80, *p* = 0.427; MX: t = −0.23, *p* = 0.820; CM: t = −0.74, *p* = 0.461). Regarding data quality, there was no difference in frame-wise displacement between the groups defined through data-driven clustering (group 1: mean = 0.01, SE = 0.001; group2: mean = 0.01, SE = 0.004, t(122) = −0.71, p = 0.480). The groups also did not differ in the number of motion outliers (group 1: mean = 18.05, SE = 1.007; group 2: mean = 17.08, SE = 1.154, t(122) = 0.63, p = 0.529). There was no significant correlation between the framewise displacement and any of the cognitive measures (Pearson correlation, MR: r = −0.12, *p* = 0.188; Vocab: r = 0.09, *p* = 0.349; DR: r = −0.00, *p* = 0.986; DM: r = −0.11, *p* = 0.226; BR: r = 0.07, *p* = 0.456; MX: r = 0.05, *p* = 0.570; CM: r = 0.11, *p* = 0.207).

Functional connectivity was assessed using independent component analysis by means of the multivariate exploratory linear decomposition into a fixed set of 25 independent components (MELODIC)^51,52^. The correspondence between the independent components derived in the data and the canonical networks described by Yeo *et al*.^53^ was established by calculating the spatial correlation between the maps. Two maps showed a high spatial correlation with the canonical default mode network (IC3: r = 0.303, IC9: r = 0.493). Two additional component maps also had a high spatial correlation with the default mode network map (r = 0.286, r = 0.276), but showed activation within the white matter and CSF and were therefore dismissed as artefacts. The default mode network in individual participants was calculated as described by Supekar and colleagues^54^. In brief, ICA with automatic dimensionality detection was performed for each participant. The groups did not differ in the number of ICA components generated (C1: mean = 100.70, SE = 1.821; C2: mean = 102.19, SE = 1.862; U = 1549, *p* = 0.254). Subsequently, a goodness-of-fit measure was calculated based on the average z-scored activation within the DMN-mask minus the activation outside of the DMN-mask and the component with the largest score was selected. To compare the spatial extent of activation within the individual DMN components between the groups, a permutation procedure with 5,000 repetitions and cluster-free threshold enhancement for correction of multiple comparisons as implemented in FSL randomise was used. To compare functional connectivity between groups, the average bandpass-filtered (0.01–0.1 Hz) signal for a 8 mm-sphere placed on the posterior cingulate cortex (PCC, MNI: 0, −52, 18), medial prefrontal cortex (mPFC, MNI: 1, 50, −5), and the left and right temporoparietal junction (TPJ, MNI: −46, −68, 32; 46, −68, 32) and the partial correlation between mPFC and PCC was calculated controlling for the signals in the left and right TPJ^55^.

### Statistical analysis

Shapiro-Wilk tests were used to test if data were normally distributed. If a significant deviation from normality was indicated (*p* > 0.05), non-parametric tests were used. Specifically, Mann-Whitney U tests were used instead of independent t-tests. The Bonferroni method was used to correct for multiple comparisons unless otherwise stated.

## Results

The analysis followed a three-step sequential logic (see Fig. 1):

- The first aim was to determine subgroups of maximally similar white matter organisation using community detection in a population-representative sample of typical development (NKI sample).
- The second aim was to investigate potential cognitive correlates of white matter tracts that distinguished groups identified in the first part of the analysis. This was carried out in large developmental samples that had detailed cognitive assessments and showed large variation in cognitive abilities (ACE & CALM sample).
- The third aim was to investigate potential driving mechanisms that distinguish the brain types by mapping the particular connections mediated by the tract that distinguished the brain types (ACE & CALM sample) and by relating differences in white matter microstructure to differences in functional connectivity (CALM sample).

### Community clustering indicates the presence of two groups that differ in FA of the cingulum

**Figure 3:**
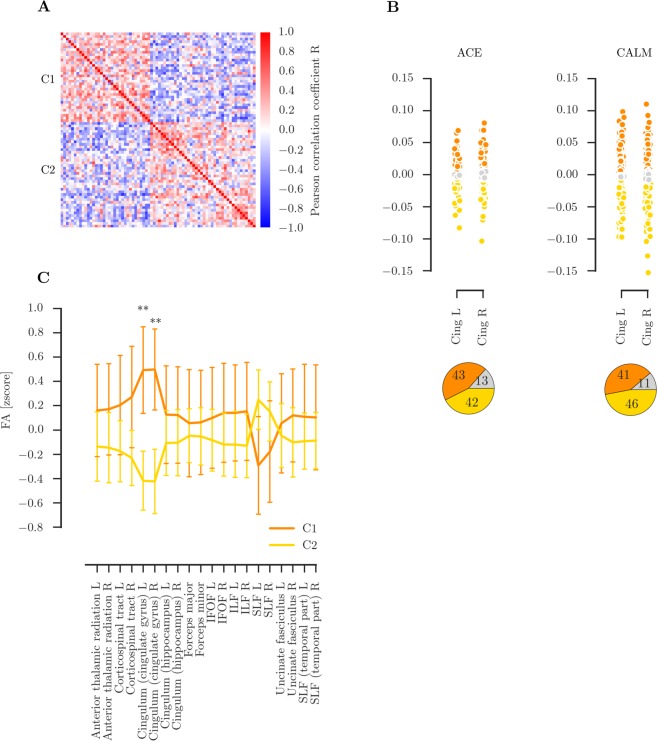
Overview of clustering based on fractional anisotropy (FA) in major white matter tracts in the NKI sample. (**A**) Correlation within groups defined through community-based clustering of FA values in the NKI sample showing two distinct groups with high correlation within the cluster. (**B**) FA values in the left and right anterior cingulum in the ACE and CALM with grouping according to the ranges identified in the NKI sample clusters. The bottom figure show the percentage of participants in each cluster range with orange for C1, yellow for C2, and grey for participants that fell outside of both ranges. (**C**) Differences in FA for each major white matter tract between the clusters identified through community clustering. The error bars show one standard error, the middle of the bar indicates the median. Significant differences between clusters were found for the left and right anterior Cingulum. Abbreviations: IFOF: inferior fronto-occipital fasciculus, ILF: inferior longitudinal fasciculus, SLF: superior longitudinal fasciculus.

Grouping children in the NKI sample according to their similarity of FA values within major white matter tracts using consensus clustering indicated the presence of two groups. The quality index indicated a good separation between groups (Q = 0.47, Fig. 3A,B). The groups defined through community clustering did not differ in age (C1: n = 34, mean = 13.568, SE = 0.59; C2: n = 40, mean = 14.23, SE = 0.470; t-test: t(72) = −0.89, p = 0.375; Equivalence test: t_lower_(65.94) = 1.355, *p* = 0.09, t_upper_(65.94), *p* = 0.001 with δ = 1.684) and did also not differ from the whole sample in proportion of males and females (C1: 24/10 male/female, χ^2^ = 0.06, *p* = 0.800; C2: 21/19, χ^2^ = 0.05, *p* = 0.825). Comparison of group assignment with an alternative clustering method (Kernighan-Lin algorithm) indicated very high agreement with an equal number of clusters using both methods and only 6 out 74 children being assigned to a different cluster depending on the algorithm. Agglomerative clustering as an alternative clustering approach provided the same results as the consensus community detection algorithm with two clusters across different linkage definitions (Ward, average, maximal, single).

Next, FA values for all tracts were compared between the groups defined through community clustering. The groups differed on the FA of the left and right anterior cingulum (cingulate gyrus region) (left anterior cingulum: C1: mean = 0.49, SE = 0.179, C2: mean = −0.42, SE = 0.121, Man-Whitney: U = 304, *p* < 0.001, *p*_corrected_ < 0.001; right anterior cingulum: mean = 0.50, SE = 0.167, C2: mean = −0.42, SE = 0.132 Man-Whitney: U = 300, *p* < 0.001, *p*_corrected_ < 0.001, see Supplementary Information Table S1 for raw FA values). There were no significant differences between the groups for any other tract (all other p_corrected_ > 0.1, see Fig. 3C). To establish the potential influence of crossing fibres, we repeated the analysis with GFA values. This comparison indicated a significantly lower GFA in C2 for the left and right anterior cingulum (left: C1: mean = 0.20, SE = 0.004, C2: mean = 0.19, SE = 0.003, U = 426, *p* = 0.003, *p*_corrected_ = 0.03, right: C1: mean = 0.22, SE = 0.004, C2: mean = 0.21, SE = 0.003, U = 430, *p* = 0.003, *p*_corrected_ = 0.034). There were no significant differences in GFA for any other tract (all other *p*_corrected_ > 0.1).

The range of age-regressed, *z*-transformed values for the left and right cingulum in each group was used to group children in the CALM and ACE sample (see Fig. 3D). A majority of the samples fell within the range for C1 or C2 (ACE: C1: n = 36 (42%), C2: n = 43 (51%), not categorized: n = 5 (6%); CALM: C1: n = 95 (49%), C2: n = 87 (45%), not categorized: n = 11 (6%), see Supplementary Information Table S2 and S3 for raw FA values). The proportion of participants being assigned to C1 or C2 was equal (ACE: *χ*^2^ = 0.64, *p* = 0.423; CALM: *χ*^2^ = 0.01, *p* = 0.943). Comparison of the within-group versus between-group connection strength indicated that the grouping based on the clusters identified in the NKI sample provided a good account of the data for both the ACE and the CALM sample that significantly differed from random group assignments (ACE: intra-cluster = 64.85, *p* < 0.001; inter-cluster: −58.95, *p* = 0.001; CALM: intra-cluster = 521.17, *p* < 0.001; inter-cluster = 21.10, *p* < 0.001).

### Brain-defined subgroups differ in cognitive performance

**Figure 4:**
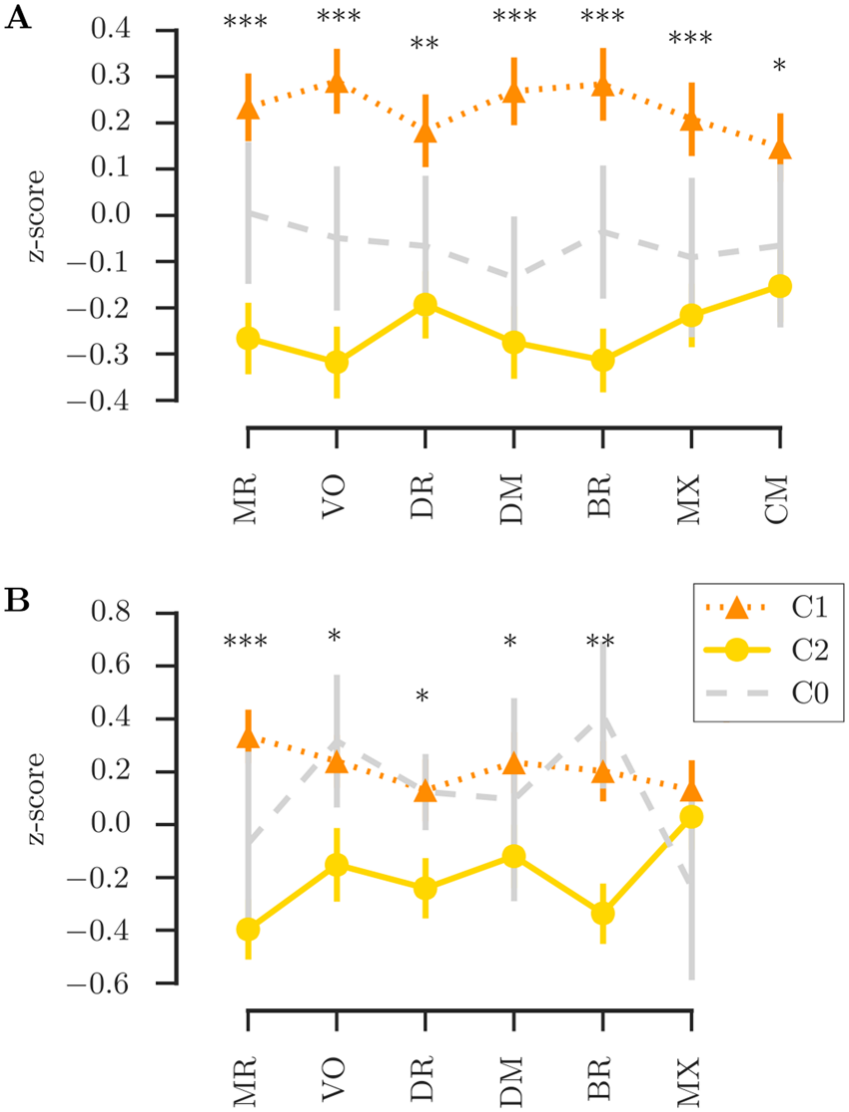
Comparison of cognitive scores between the groups defined based on cingulum FA in the CALM sample (**A**) and the ACE sample. (**B**) Legend: Comparison between C1 and C2; C0: not assigned to C1 or C2, ***p_corrected_ < 0.001, **p_corrected_ < 0.01, *p_corrected_ < 0.05.

Next, differences in cognitive performance between the clustering-defined groups were investigated. For the CALM sample, children who fell within the C1 range showed significantly higher performance across all cognitive assessments (all *p*_corrected_ < 0.05, see Fig. 4A). For the ACE sample, C1 showed significantly higher performance across all cognitive measures, apart from visuospatial working memory (*p* = 0.207, *p*_corrected_ > 0.999, see Fig. 4B).

We further investigated the reliability of the clustering-defined grouping for predicting behavioural performance. For the CALM sample, predictive performance in unseen data was above chance for scores on Matrix Reasoning, Vocabulary, Mr. X, and Delayed Story Recall with prediction accuracy between 60 and 75% (see Table 1). The optimal percentile cut-off identified in the training data set and the training performance are listed in the columns *%ile* and *train*. The fraction of participants who were accurately predicted to score above or below the median of all cases based on their group assignment is listed for the train and test set column. A statistical comparison to random assignment (1000 permutations) is listed in the column labelled *p*. For the ACE sample, there was above chance prediction for unseen data for Matrix Reasoning, Vocabulary, Backward Digit Recall, and Mr. X with prediction accuracies around 70% (see Table 2).

**Table 2:** Generalizability of behavioural predictions based on clustering-defined groups.

	CALM	%ile	train	test	*p*	ACE	%ile	train	test	*p*
Matrix Reasoning		50	0.62	0.74	0.003		50	0.65	0.71	0.027
Vocabulary		65	0.68	0.64	0.028		55	0.89	0.72	0.025
Digit Recall		65	0.60	0.56	0.214		60	0.61	0.67	0.089
Dot Matrix		50	0.63	0.56	0.214		70	0.61	0.50	0.387
Backward Digit Recall		50	0.62	0.58	0.214		50	0.71	0.77	0.025
Mr. X		50	0.60	0.69	0.004		70	0.5	0.71	0.025
Delayed Story Recall		50	0.62	0.64	0.028		N/A	N/A	N/A	N/A

The optimal percentile cut-off identified in the training data set and the training performance are listed in the columns %ile and train. The fraction of participants who were accurately predicted to score above or below the median of all cases based on their group assignment is listed for the train and test set column. A statistical comparison to random assignment (1000 permutations) is listed in the column labelled *p*.

### Subgroups show differences in fronto-parietal and fronto-temporal connections of the cingulum

**Figure 5:**
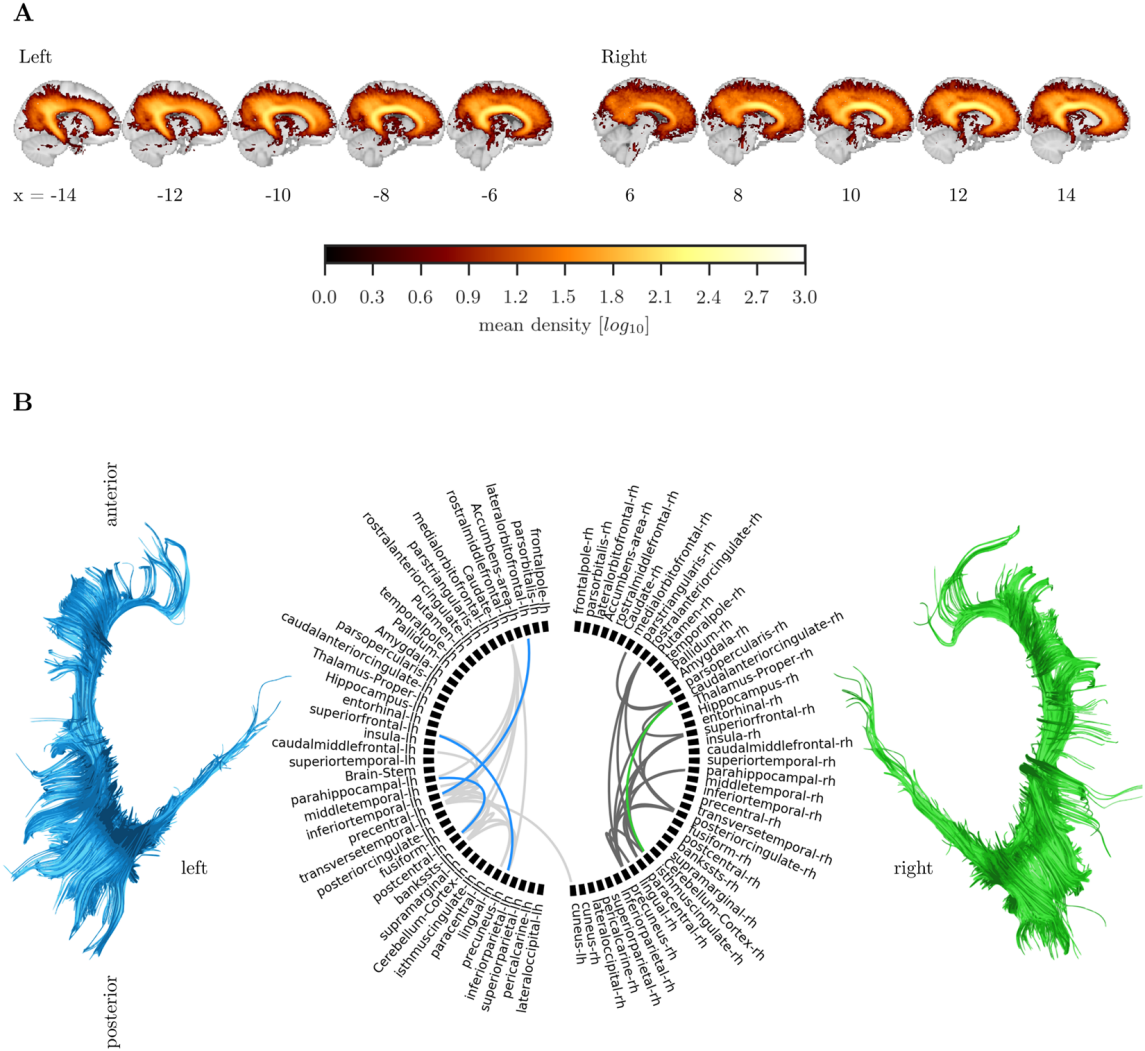
Structural connectivity of the cingulum. (**A**) Average streamline density of the cingulum reconstruction. (**B**) Connections of the cingulum. Grey lines indicate connections that were present in at least 60% of the sample. Blues lines indicate connections of the left cingulum that had fewer streamlines in C2 compared to C1 and green lines indicate fewer connections in C2 compared to C1 for the right cingulum.

The groups were compared on the number of streamlines within the cingulum that connect cortical and subcortical regions (ACE & CALM sample). Streamlines associated with the cingulum were densest around in the core area of the cingulum, but also extended into the frontal, parietal, and temporal cortex in both hemispheres (see Fig. 5A). Areas that were consistently connected through streamlines of the cingulum included the orbitofrontal and superior frontal cortex, insula, inferior and superior parietal cortex, precuneus, and parahippocampal cortex (see Fig. 5B). The summed streamline density was higher in the left cingulum compared to the right cingulum (left: mean = 53.34, SE = 0.449; right: mean = 45.80, SE = 0.435, U = 7959, *p* < 0.001). The total number of connections was also higher in the left compared to the right cingulum (left: mean = 44.10, SE = 0.355; right: 35.31, SE = 0.302, U = 3282, *p* < 0.001). Differences between groups in individual cingulum-mediated connections were selected using a cross-validation procedure, which indicated significant differences between the brain types for some cingulum connections that were also confirmed in a hold-out sample (20%). The groups showed consistent differences in the connections between the precuneus and superior frontal cortex, inferior temporal cortex and lateral orbitofrontal cortex, parahippocampal cortex and post-central cortex in the left hemisphere, and the anterior cingulate cortex and posterior cingulate cortex in the right hemisphere (see Fig. 5B). C2 had had lower streamline density in all of these connections compared to C1.

### Relationship between structural connectivity and default mode network activation

**Figure 6:**
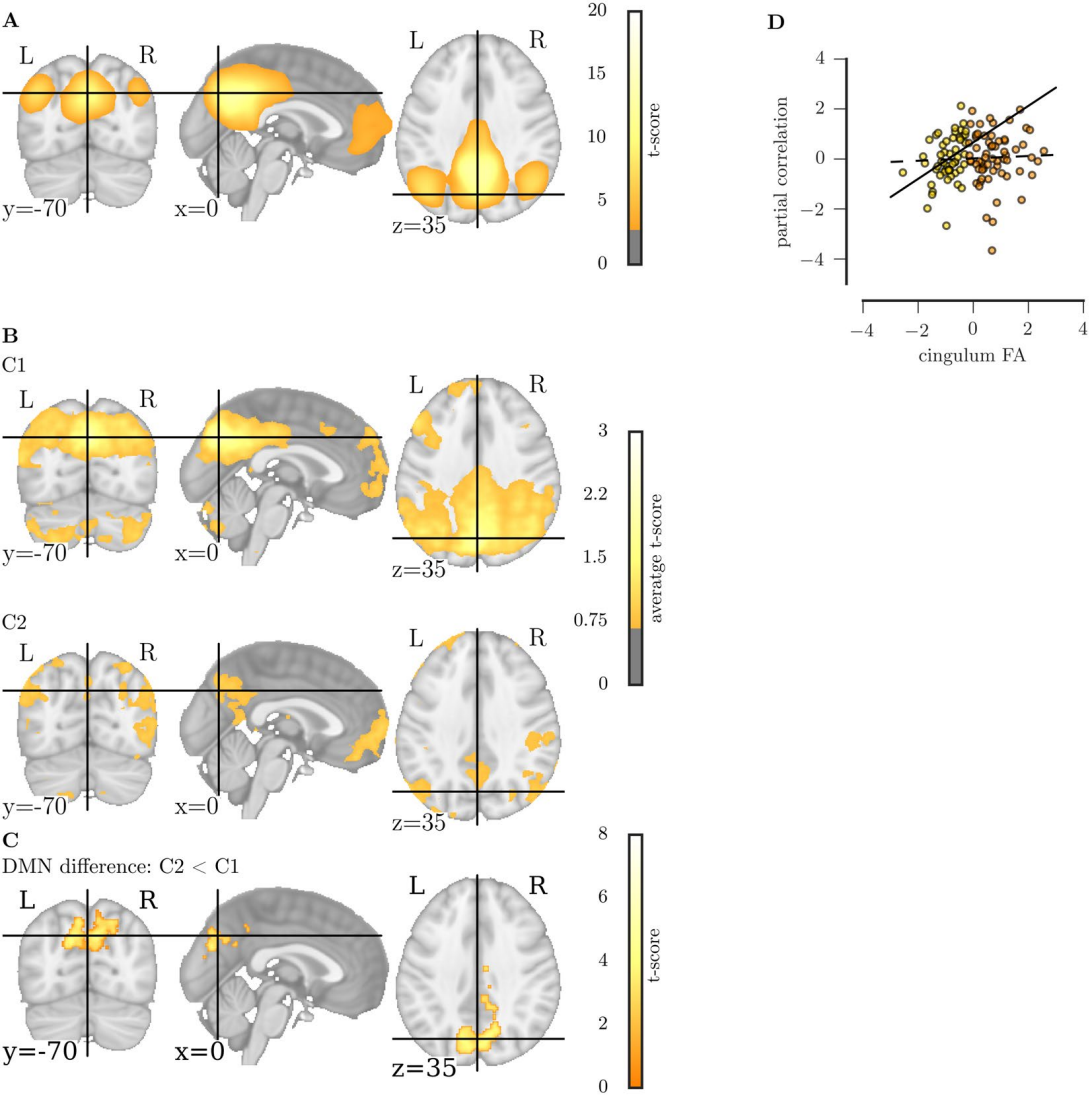
(**A**) Default mode network (DMN) at the group level, (**B**) individual DMN average in C1 and C2. (**C**) Results of a two-sample t-test contrasting the DMN in C1 and C2 showing a reduced spatial extent of C1 compared to C2 in the posterior cingulate cortex. (**D**) Relationship between the partial correlation of the medial prefrontal cortex and posterior cingulate cortex and FA of the cingulum in C1 and C2.

The default mode network (DMN) could be identified in group-level ICA (CALM sample, see Fig. 6A). The groups did not differ in the number of components identified in individual independent component decomposition (C1: mean = 100.70, SE = 1.821; C2: mean = 102.19, SE = 1.862; U = 1549, *p* = 0.254). Comparison of ICA-derived maps between C1 and C2 indicated that the spatial extent of the DMN was significantly reduced in the posterior cingulate cortex for C1 (MNI peak coordinates: 0, −70, 35; TCFE-corrected *p* < 0.05; see Fig. 6C). Next, we investigated the relationship between variation in the microstructure of the cingulum and the strength of functional connectivity between the medial prefrontal cortex (mPFC) and posterior cingulate cortex (PCC). While the clustering-defined groups did not differ in absolute mPFC-PCC partial correlation (C1: mean = 0.31, SE = 0.020; C2: mean = 0.32, SE = 0.020, U = 1511, *p* = 0.239), analysis of the relationship between cingulum FA and mPFC-PCC correlation indicated a significant group interaction (group x cingulum FA: *t*(118) = 2.01, *p* = 0.047). Follow-up analyses showed that cingulum FA was significantly associated with the mPFC-PCC correlation in C1 (beta = 0.38, *p* = 0.005), but not in C2 (beta = 0.03, *p* = 0.811, see Fig. 6D).

## Discussion

The first aim of the study was to test whether it is possible to identify groups on the basis of individual differences in white matter organization. We employed data-driven community clustering to create subgroups of participants that were maximally similar in white matter organisation across 20 major white matter tracts. In theory, this could have resulted in multiple groups with a highly complex differentiation of brain organization. However, the subgrouping is dominated by the integrity of a pair of large tracts within the brain: the results indicated the presence of two groups primarily distinguished by FA of the left and right cingulum. Whilst this finding is somewhat unexpected, the cingulum has been implicated in a broad range of psychiatric and developmental disorders, including autism spectrum disorder^56^, attention deficit hyperactivity disorder^57^, schizophrenia^58^, major depression^59^, mild cognitive impairment^60^, and dementia^61^. There are several possible reasons why the cingulum may play a central role in individual differences in neurocognitive development. One explanation may lie in the prolonged development of the cingulum. The cingulum is one of the only tracts that show extended development with changes throughout childhood and adolescence, only reaching a stable level in the third decade of life^12,62^. Similar tracts, also involved in long-range connectivity, like the superior longitudinal fasciculus, inferior longitudinal fasciculus, and inferior fronto-occipital fasciculus already plateau by the end of the second decade of life. Consequently, individual differences may arise from differences in timing of cingulum maturation. Further, the cingulum may be particularly sensitive to environmental influences. The cingulum has been found to be particularly sensitive to neonatal complications, which, in turn, are a risk factor for unfavourable developmental outcomes later in life like a heightened risk for psychiatric disorders^63,64^. That said, diffusion properties of the cingulum have also been found to change following relatively short working memory or motor training interventions^65–67^ indicating that the individual differences in the cingulum may not necessarily be fixed throughout the lifespan. In sum, the inter-individual differences in cingulum FA identified in the current analysis may be a marker of accumulated differences in experience between individuals over their distant and recent lifetime.

The second aim of the study of the study was to investigate whether subgroups formed on the basis of similarity of white-matter organization differ in cognitive performance. Applying the cingulum-based grouping to two independent samples of typically-developing children or struggling learners showed that children with lower cingulum FA showed lower performance on assessments of general intelligence, vocabulary, and short-term, long-term, and working memory. This broad difference in cognitive performance clashes with previous findings that indicated a comparatively narrow association between cingulum FA and executive function, which included sustained and divided attention^68^, working memory^69^, and planning^70^. However, broader associations between the cingulum and cognitive abilities have been reported in different patient populations^64^. For instance, cingulum integrity was found to relate to memory function in mild cognitive impairment and Alzheimer’s disease^71–73^. Further, cingulum integrity was found to be associated with symptom severity in major depression, obsessive compulsive disorder (OCD), and post-traumatic stress disorder (PTSD)^74–76^. A potential explanation for the broad and inconsistent contribution of the cingulum to measures of cognitive performance and symptom severity is that it may support circuits involved in affect and motivation. Differences in affect and motivation can have a broad impact across measures of cognition and symptom severity. Areas thought to be central for decision making (anterior cingulate cortex, orbitofrontal cortex, medial thalamus) are at least in part connected through cingulum connections^64^. The cingulum also supports connections between limbic areas that have been linked to emotional processing^77^, which may be particularly important for effort-based decision making with broad consequences for performance on cognitive tasks^78^. Further, frontoparietal connections via the cingulum play a central role in attention^79^, which may also impact other assessments, e.g. attention directed to internal cues may affect symptom severity ratings or influence performance on other cognitive tasks.

In addition to the circuit-specific role of the cingulum, the importance of the cingulum may be linked to its role within the global white matter connectome, i.e. the cingulum may contribute to a network architecture that supports cognitive function optimally. The results of the connectomics analysis in the current study show that cingulum contains extensive short-range and long-range connections between cortical areas in each hemisphere. Further, the analysis of default mode network activation shows that the cingulum is important for functional integration between anterior and posterior parts (medial prefrontal cortex and posterior cingulate cortex) of this distributed cortical network. While a strong association between cingulum FA and mPFC-PCC was observed in a group of children with low cingulum FA, there was no association in children with high cingulum FA. A possible interpretation is that low cingulum FA limits the communication between these regions, but only up to a critical value. Once this critical value is reached – as in the case of the high FA group - further increases no longer influence the strength of the functional connection. Integration between parietal and prefrontal areas is central to some theories of general intelligence^80,81^. According to the parietal-prefrontal integration theory (P-FIT), general cognitive ability arises from the interplay between parietal areas involved in the integration and abstraction of sensory information, prefrontal areas involved in reasoning and problem-solving, and the anterior cingulate cortex involved in response selection^82,83^. The role of the cingulum may be to enable the efficient communication between these regions. However, the connectivity mapping in the current study indicates that the cingulum plays a broader role in structural connectivity beyond connections along the prefrontal-parietal axis. The dense short-range and longer-range connections of the cingulum may enable communication within multiple large-scale structural brain networks. Indeed, the connections implicated in the group difference involved the prefrontal and posterior parietal areas that are part of a small number of so-called hub regions that are densely connected in functional and structural brain networks^84^. The connectivity of these hub regions is thought to form the backbone of a network architecture that enables the efficient transfer of information and the rapid transition between different network states, both of which may be necessary for cognitive performance in complex tasks^81^.

There are some limitations to the current study. First, this study explored variation in white matter microstructure in pre-collected samples and was limited to the available data. Therefore, it was not possible to investigate if genetic or environmental factors, such as socio-economic background, contribute differentially to white matter microstructure in the brain types identified in the analysis. Further, the samples differed in their age range and gender distribution. Therefore, we focused on identifying brain types that are not influenced by these factors by statistically controlling for the influence of age and gender. Given the well-documented development of white matter through childhood and adolescent, it is likely that different groupings may dominate at particular stages of neurocognitive development. While it was not possible to adequately address this in the current study, it will be possible to investigate developmental effects when large-scale, longitudinal samples become available in the near future.

Another important caveat is that the grouping identified in the current study depends on the data used to inform the grouping and the definition of similarity. Just like there are multiple valid ways to characterize individual differences between people, e.g. short vs tall, young vs old, male vs female, there are multiple ways to describe individual differences in neurocognitive development. Previously identified groupings based on behavioural features^9^ may therefore not necessarily fully align with the white matter-based grouping identified here, even though they seem to converge on similar white matter differences, i.e. fronto-parietal connectivity. Employing a data-driven grouping has the advantage of creating groups that are maximally distinct and maximally homogeneous, which can be a powerful approach to investigate the drivers of individual differences as showcased in this analysis. Further, the homogeneity within groups is formally defined and quantified, which makes group-based analyses more objective than through traditional top-down definitions. However, there is currently no general agreement which metric captures individual differences the best, e.g. correlation-based vs distance-based metrics. The same applies to the plethora of clustering algorithms, e.g. centroid-based, hierarchical, density-based, network-based etc. Here, we focused on a methodological approach that has been previously employed for analysis at the behavioural and cognitive level^7–9^. However, this is by no means the only approach for data-driven grouping and other approaches have produced highly promising results, e.g. see^85^. Future research based on population-representative samples with rich phenotypic characterisation will need to establish which definition of similarity and grouping is most useful to inform our understanding of individual differences in neuroanatomy. Further, the current analysis only investigates individual differences in brain anatomy at one coarse anatomical scale. Future studies could employ data-driven methods to identify if these individual differences are apparent at different scales, e.g. through fractal topological clustering^86^.

In conclusion, this is the first study to investigate the relationship between individual differences in white matter connections and cognitive performance with a brain-first approach. The results indicate that consistent individual differences exist in the microstructural organization of the left and right cingulum. Lower microstructural organisation of the cingulum was related to lower cognitive performance across a range of cognitive domains and was also linked to reduced BOLD activation within the default mode network. These findings suggest that cingulum connections play an important role in brain organisation and cognitive performance in childhood and adolescence. The current study is an initial step towards a bigger goal of understanding individual differences in neuroanatomy; it illustrates the benefits of bringing together large samples, multi-modal characterisation, and machine learning approaches like community clustering to identify biologically-defined groups that are difficult to distinguish at a behavioural level. In future, this new approach may enable our field to isolate key biological mechanism that drive differences in brain organisation, allowing for more objective identification of individual needs, and open new avenues for effective interventions.

## Supplementary information

Supplementary Information
