# Supplementary material for: The cingulum as a marker of individual differences in neurocognitive development
Source: Sci Rep. 2019 Feb 19;9:2281. doi: 10.1038/s41598-019-38894-z (PMC6381161; doi:10.1038/s41598-019-38894-z)
Supplement: Supplementary file 1 — Supplementary Information [file 41598_2019_38894_MOESM1_ESM.docx]

**The cingulum as a marker of individual differences in neurocognitive development**

Supplementary Information

Joe Bathelt^1*^, Amy Johnson^1^, Mengya Zhang^1^, & Duncan E. Astle^1^

Supplementary Information

**Table S1:** Raw FA values of clustering-defined groups in the NKI sample

|  | C1 | | C2 | |
| --- | --- | --- | --- | --- |
| Tract | mean | SE | mean | SE |
| Anterior thalamic radiation L | 0.34 | 0.008 | 0.33 | 0.006 |
| Anterior thalamic radiation R | 0.33 | 0.008 | 0.32 | 0.006 |
| Corticospinal tract L | 0.48 | 0.011 | 0.46 | 0.007 |
| Corticospinal tract R | 0.48 | 0.011 | 0.47 | 0.008 |
| Cingulum (cingulate gyrus) L | 0.43 | 0.015 | 0.40 | 0.009 |
| Cingulum (cingulate gyrus) R | 0.37 | 0.014 | 0.34 | 0.007 |
| Cingulum (hippocampus) L | 0.30 | 0.009 | 0.26 | 0.006 |
| Cingulum (hippocampus) R | 0.34 | 0.009 | 0.28 | 0.007 |
| Forceps major | 0.50 | 0.012 | 0.49 | 0.006 |
| Forceps minor | 0.37 | 0.01 | 0.36 | 0.006 |
| IFOF L | 0.38 | 0.009 | 0.37 | 0.006 |
| IFOF R | 0.39 | 0.009 | 0.38 | 0.006 |
| ILF L | 0.37 | 0.008 | 0.36 | 0.006 |
| ILF R | 0.39 | 0.009 | 0.37 | 0.006 |
| SLF L | 0.34 | 0.007 | 0.33 | 0.005 |
| SLF R | 0.36 | 0.007 | 0.35 | 0.005 |
| Uncinate fasciculus L | 0.35 | 0.011 | 0.34 | 0.006 |
| Uncinate fasciculus R | 0.34 | 0.01 | 0.34 | 0.005 |
| SLF (temporal part) L | 0.41 | 0.01 | 0.44 | 0.006 |
| SLF (temporal part) R | 0.49 | 0.013 | 0.51 | 0.007 |

**Table S2:** Raw FA values of groups in the CALM sample

|  | C1 | | C2 | |
| --- | --- | --- | --- | --- |
| Tract | mean | SE | mean | SE |
| Anterior thalamic radiation L | 0.40 | 0.002 | 0.38 | 0.002 |
| Anterior thalamic radiation R | 0.39 | 0.002 | 0.37 | 0.002 |
| Corticospinal tract L | 0.56 | 0.002 | 0.54 | 0.002 |
| Corticospinal tract R | 0.56 | 0.002 | 0.54 | 0.002 |
| Cingulum (cingulate gyrus) L | 0.53 | 0.002 | 0.46 | 0.003 |
| Cingulum (cingulate gyrus) R | 0.46 | 0.003 | 0.39 | 0.004 |
| Cingulum (hippocampus) L | 0.37 | 0.004 | 0.32 | 0.005 |
| Cingulum (hippocampus) R | 0.39 | 0.003 | 0.35 | 0.003 |
| Forceps major | 0.57 | 0.002 | 0.54 | 0.002 |
| Forceps minor | 0.45 | 0.002 | 0.43 | 0.001 |
| IFOF L | 0.46 | 0.002 | 0.43 | 0.002 |
| IFOF R | 0.46 | 0.002 | 0.44 | 0.002 |
| ILF L | 0.44 | 0.002 | 0.41 | 0.002 |
| ILF R | 0.46 | 0.002 | 0.43 | 0.002 |
| SLF L | 0.40 | 0.002 | 0.37 | 0.002 |
| SLF R | 0.41 | 0.002 | 0.38 | 0.002 |
| Uncinate fasciculus L | 0.42 | 0.002 | 0.39 | 0.003 |
| Uncinate fasciculus R | 0.41 | 0.002 | 0.37 | 0.004 |
| SLF (temporal part) L | 0.49 | 0.003 | 0.46 | 0.003 |
| SLF (temporal part) R | 0.55 | 0.003 | 0.53 | 0.003 |

**Table S3**: Raw FA values of groups in the ACE sample

|  | C1 | | C2 | |
| --- | --- | --- | --- | --- |
| Tract | mean | SE | mean | SE |
| Anterior thalamic radiation L | 0.40 | 0.001 | 0.39 | 0.002 |
| Anterior thalamic radiation R | 0.39 | 0.001 | 0.37 | 0.002 |
| Corticospinal tract L | 0.56 | 0.002 | 0.54 | 0.003 |
| Corticospinal tract R | 0.57 | 0.002 | 0.54 | 0.005 |
| Cingulum (cingulate gyrus) L | 0.53 | 0.002 | 0.47 | 0.005 |
| Cingulum (cingulate gyrus) R | 0.46 | 0.003 | 0.4 | 0.004 |
| Cingulum (hippocampus) L | 0.37 | 0.004 | 0.33 | 0.006 |
| Cingulum (hippocampus) R | 0.39 | 0.004 | 0.37 | 0.005 |
| Forceps major | 0.57 | 0.002 | 0.54 | 0.006 |
| Forceps minor | 0.45 | 0.001 | 0.43 | 0.002 |
| IFOF L | 0.46 | 0.001 | 0.44 | 0.003 |
| IFOF R | 0.46 | 0.002 | 0.44 | 0.004 |
| ILF L | 0.44 | 0.002 | 0.42 | 0.004 |
| ILF R | 0.46 | 0.002 | 0.43 | 0.005 |
| SLF L | 0.40 | 0.001 | 0.38 | 0.003 |
| SLF R | 0.41 | 0.001 | 0.39 | 0.004 |
| Uncinate fasciculus L | 0.43 | 0.002 | 0.39 | 0.006 |
| Uncinate fasciculus R | 0.41 | 0.002 | 0.37 | 0.005 |
| SLF (temporal part) L | 0.48 | 0.004 | 0.47 | 0.006 |
| SLF (temporal part) R | 0.55 | 0.004 | 0.54 | 0.005 |
